# Macular vascular features of different types of diabetic macular edema using ocular coherence tomography angiography- a comparative study

**DOI:** 10.1186/s40942-023-00469-6

**Published:** 2023-05-18

**Authors:** Ghada A. Nassar, Ibrahim M. Maqboul, Ayman Gehad El-Nahry, Lameece Moustafa Hassan, Ahmed B. Shalash

**Affiliations:** Cairo University, 3 Road 217, Degla Maadi, Cairo, Egypt

**Keywords:** Diabetic macular edema, Cystoid macular edema, Diffuse retinal thickening, Optical coherence tomography angiography, Foveal avascular zone, Choriocapillaris, Neurosensory detachment

## Abstract

**Background:**

To compare the microvascular features of different subtypes of diabetic macular edema (DME) by optical coherence tomography angiography (OCTA).

**Methods:**

A cross-sectional study including treatment-naive patients with DME. Eyes were divided according to optical coherence tomography determined morphology into two groups: cystoid macular edema (CME) and diffuse retinal thickening (DRT), with further subdivision according to the presence of subretinal fluid. All patients underwent 3 × 3 and 6 × 6 mm OCTA scans of the macula to compare the foveal avascular zone (FAZ) area, vascular density (VD) of the superficial (SCP) and deep (DCP) capillary plexus and choriocapillaris flow (CF). Laboratory findings (HbA1C and triglyceride levels) were also correlated with the OCTA findings.

**Results:**

The study included 52 eyes, 27 had CME and 25 had DRT. There were no significant differences between the VD of the SCP (p = 0.684) and DCP (p = 0.437), FAZ of SCP (p = 0.574), FAZ of DCP (p = 0.563) and CF (p = 0.311). Linear regression analysis revealed that DME morphology was the strongest predictor for BCVA. Other significant predictors included HbA1C and triglyceride levels.

**Conclusion:**

The morphology of DME, irrespective of SRF, was most significantly correlated with BCVA in treatment-naive patients and CME subtype could be an independent predictor of poor BCVA in patients with DME.

## Background

Diabetic macular edema (DME) is the most prevalent sight-threatening complication of diabetic retinopathy (DR) in developed countries, especially in patients with type II diabetes mellitus (DM) [1]. It was estimated that the global prevalence of DME in both types of DM was approximately 7.48% [2].

By optical coherence tomography (OCT) there are three patterns or subtypes of DME, which can occur simultaneously in the same eye. They include: cystoid macular edema (CME), diffuse retinal thickening (DRT), and subretinal fluid (SRF) [3].

The OCT patterns of DME can be prognostic factors in the response to treatment, thus indicating differences in the underlying pathophysiology of each subtypes [4].

Optical coherence tomography angiography (OCTA) is a new non-invasive technique of ocular angiography based on OCT technology, which can measure vascular density and detect changes in DR, such as nonperfusion areas, microaneurysms, IRMA, or neovascularization [5].

In this study, we attempted to compare the macular microvascular features of different subtypes of DME, using OCTA, to determine their underlying structural features and whether these features may contribute to a different underlying pathophysiology. We also correlated these OCTA features with clinical and laboratory findings.

## Methods

This cross-sectional observational study was performed at the Ophthalmology department of the Cairo University hospital, between August 2021 and February 2022. It was approved by the Cairo University research ethics committee code 385/2021 and followed the tenets of of the Declaration of Helsinki. A written informed consent was obtained from each patient.

The study included any patients with type 2 DM, older than 18 years of age, with DME by OCT (Optovue, Inc, Fremont, CA). DME was defined as a central macular thickness (CMT) of more than 300 μm with evidence of edema by OCT. Eyes were then subdivided into CME or DRT, with or without SRF. CME was defined by the presence of predominantly cystoid hyporeflective spaces in the macula, while DRT was defined by the presence of predominantly diffuse outer macular edema. SRF was defined by the presence of hyporeflective fluid underneath the neurosensory retina. All grades of DR were eligible for inclusion.

Exclusion criteria included mixed types of DME, which could not be classified as predominantly either CME or DR, eyes with SRF only (without increased CMT), history of other retinal diseases that could affect macular perfusion (i.e. retinal vein occlusion and central serous chorioretinopathy) and history of treatment for DME. Other exclusion criteria included concomitant ocular conditions such as glaucoma and uveitis, eyes with major imaging artifacts or large segmentation errors on OCTA that could not be corrected, high myopia, presence of an epiretinal membrane or vitreomacular traction, cataract surgery within 6 months and previous vitreoretinal surgeries.

Each patient underwent a complete ophthalmic examination including best corrected visual acuity (BCVA), intraocular pressure measurement, slit-lamp biomicroscopy, and fundus examination. The duration of DM and its control through HbA1C measurement, kidney functiont tests, and lipid profile were also recorded for each patient.

### Acquisition and analysis of OCTA images

Spectal domain OCTA was performed using the 3 × 3 and 6 × 6 mm macular scans of the RTVue XR Avanti (Optovue, Inc, Fremont, CA). The vascular density (VD) of the superficial (SCP) and deep capillary plexuses (DCP) was determined in the whole image, para and parafoveal regions. Automatic segmentation divided the intraretinal layers into superficial capillary plexus (3 μm below ILM to 15 μm below IPL), deep capillary plexus (15–70 μm below IPL), outer retina (70 μm below IPL–30 μm below retinal pigment epithelium, RPE, reference) and choroidal capillary (30 μm–60 μm below RPE reference). Two images, one for the superficial capillary plexus and one for the deep capillary plexus, were taken for each eye.

The subfoveal choriocapillaris flow area (SCFA) was measured using the circle contour of the built-in flow function.

**Fig. 1:**
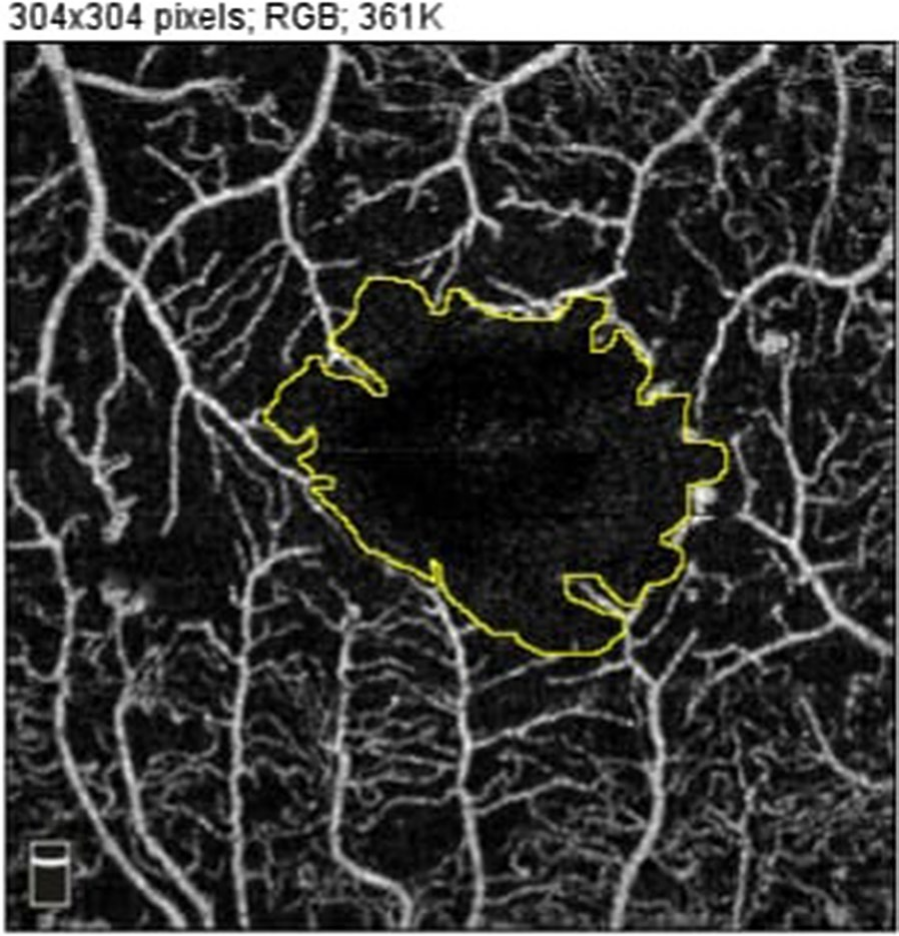
Manual measurement of FAZ using ImageJ software program

The foveal avascular zone (FAZ) area was measured in millimeters square manually in the SCP and DCP using the freehand tool of ImageJ (by connecting the points along the termination of the capillary network in the parafoveal area) separately by two masked consultants (AGN and GA) and an average value was taken. (National Institutes of Health, Bethesda, Maryland, USA) (Fig. 1).

Scans were repeated if there was an insufficient signal strength index (SSI; < 5), presence of blink artifacts, poor fixation leading to motion or doubling artifacts, areas of localized signal loss from media opacity, or major segmentation errors. Minor segmentation errors were corrected manually using the built-in machine software. All investigative data was recorded and compared between the different groups.

### Statistical analysis

Data was coded and entered using the statistical package for the Social Sciences (SPSS) version 28 (IBM Corp., Armonk, NY, USA). Data was summarised using mean and standard deviation for quantitative variables and frequencies (number of cases) and relative frequencies (percentages) for categorical variables. Comparisons between groups were done using unpaired t test for 2 groups and analysis of variance (ANOVA) with multiple comparisons post hoc test when comparing more than 2 groups. For comparing categorical data, Chi square (χ2) test was performed. Exact test was used instead when the expected frequency is less than 5. Correlations between quantitative variables were done using Pearson correlation coefficient. Linear regression analysis was done to predict VA using significant parameters***.*** P-values less than 0.05 were considered as statistically significant.

## Results

### Clinical and demographic data

Fifty-two eyes of 34 patients were included in the study; 27 in the CME group, and 25 in DRT group. The mean age of patients was 56.17 ± 7.79 years and 20 (29 eyes) were women. All patients had type 2 diabetes. The mean best-corrected visual acuity (BCVA) was 0.67 ± 0.29, with a mean HbA1c of 9.50 ± 1.81%. Clinical and laboratory characteristics both groups were not significantly different (Tables 1 and 2).

**Table 1:** Clinical and demographic data of patients

Criteria	Count
Groups (n & %)	
CME/SFF−−	17, 32.7%
CME/SRF + +	10, 19.2%
DRT/SRF−−	15, 28.8%
DRT/SRF + +	10, 19.2%
Age	56.17 ± 7.79
Sex M/ F (n, %)	23 (44.2%)/29 (55.8%)
HTN (n, %)	29, 55.8%
DR severity	
Mild NPDR	3, 5.8%
Moderate NPDR	16, 30.8%
Severe NPDR	18, 34.6%
PDR	15, 28.8%
IOP (mm Hg ± SD)	15.25 ± 3.04
BCVA (LogMAR ± SD)	0.67 ± 0.29
HbA1c (n ± SD)	9.50 ± 1.81
Duration of DM (years)	10.25 ± 2.48

*n* Number, *CME* Cystoid Macular edema, *DRT* Diffuse Retinal Thickening, *SRF* Subretinal Fluid, *M* Male, *F* Female, *DM* Diabetes Mellitus, *HTN* Hypertension, DR Diabetic Retinopathy, *NPDR* Non Proliferative Diabetic Retinopathy, *PDR* Proliferative Diabetic Retinopathy, *IOP* Intraocular Pressure, *SD* Standard Deviation, *BCVA* Best Corrected Visual Acuity, *HbA1C* Glycosylated Haemoglobin

**Table 2:** Demographic and laboratory characteristics of both groups (CME Vs DRT)

	CME group	DRT group	P value
Number of cases	27	25	
Mean age (years)	56.70 ± 7.87	55.60 ± 7.81	0.614
Sex, M/F	13/14	9/16	0.250
Severity of DR n (%)			
Mild NPDR	1 (3.7%)	2 (8.0%)	0.454
Moderate NPDR	8 (29.6%)	8 (32.0%)	
Severe NPDR	12(44.4%)	6 (24.0%)	
PDR	6(22.2%)	9 (36.0%)	
Duration of DM (years)	9.93 ± 2.43	10.68 ± 2.50	0.347
IOP (mm Hg)	15.3 ± 2.87	15.2 ± 3.21	0.8
HTN, n (%)	15 (55.7%)	16 (56%)	0.974
BCVA (LogMAR)	0.88 ± 0.29	0.64 ± 0.22	0.001*
HbA1c	9.38 ± 1.84	9.62 ± 1.80	0.640
Kidney function (± SD)			
Creatinine	1.08 ± 0.28	0.98 ± 0.29	0.203
Urea	38.31 ± 11.02	39.25 ± 8.94	0.739
Lipid profile (± SD)			
Cholesterol	231.26 ± 47.89	239.60 ± 58.45	0.575
TCG	186.15 ± 105.33	195.72 ± 112.13	0.752
HDL	45.73 ± 7.68	46.67 ± 9.89	0.703
LDL	153.78 ± 48.58	170.84 ± 54.10	0.237
Presence of SRF (%)	37.03%	40%	

*n* Number, *CME* Cystoid Macular edema, *DRT* Diffuse Retinal Thickening, *SRF* Subretinal Fluid, *M* Male, *F* Female, *DM* Diabetes Mellitus, *HTN* Hypertension, *DR* Diabetic Retinopathy, *NPDR* Non Proliferative Diabetic Retinopathy, *PDR* Proliferative Diabetic Retinopathy, *IOP* Intraocular Pressure, *SD* Standard Deviation, *BCVA* Best Corrected Visual Acuity, *HbA1C* Glycosylated Haemoglobin, *TCG* Triglycerides, *HDL* High density lipoprotein, *LDL* Low density lipoprotein

*Highly significant comparison

### OCT and OCTA characteristics of both groups (CME Vs DRT)

OCTA parameters did not significantly differ between the two groups including: VD in SCP (p = 0.684) and DCP (p = 0.437), FAZ of SCP (p = 0.574), FAZ of DCP (p = 0.563), and the SCFA (p = 0.311). Only the logMAR BCVA showed a significant difference (p = 0.001) between both groups, being significantly worse in the CME group (Table 1).

**Fig. 2:**
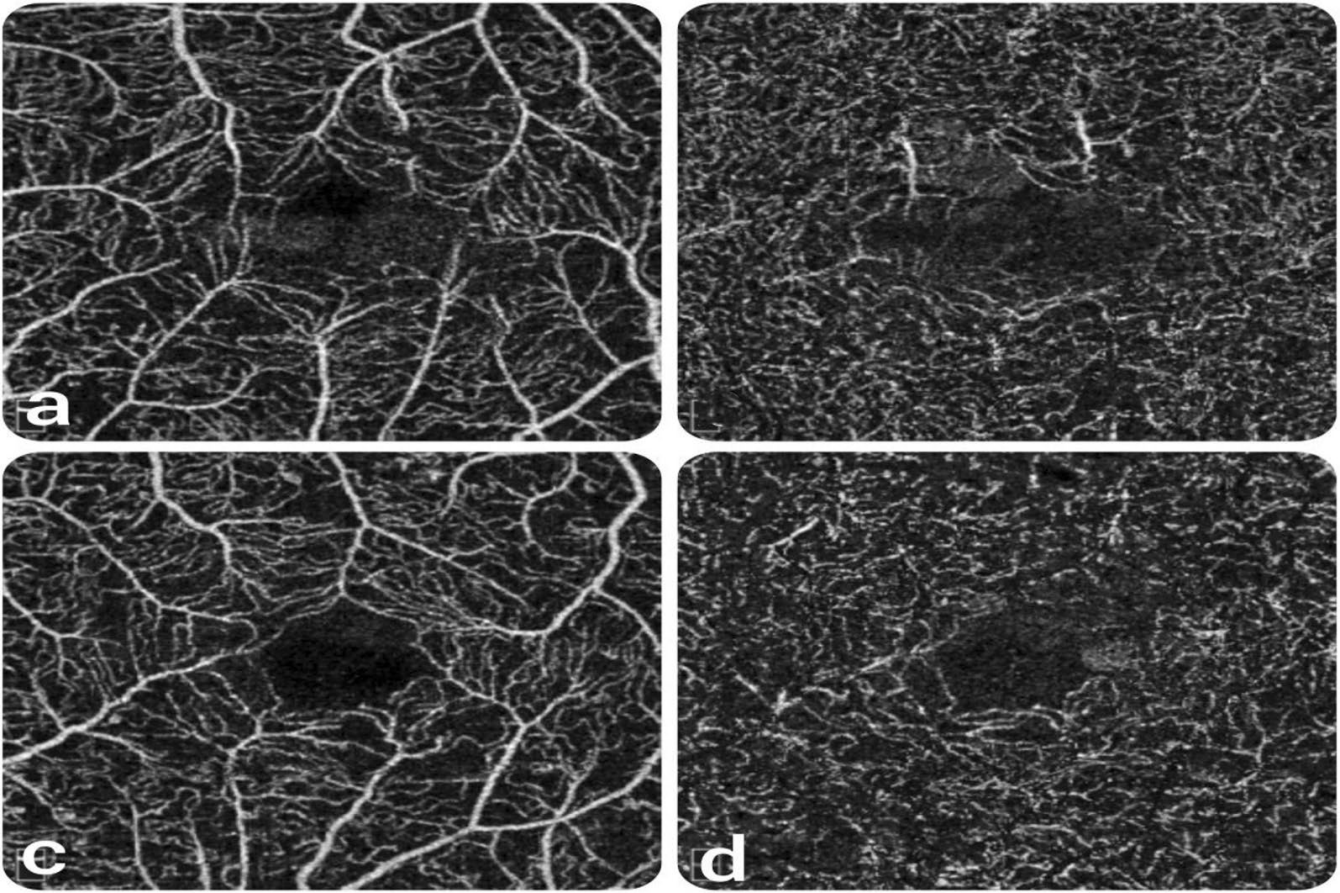
FAZ measurements on 3 × 3 OCTA imaging in different morphological patterns of DME (**a**); shows FAZ in SCP in CME Patient N.7, (**b**); shows FAZ in DCP in CME of same patient in (**a**), **c** shows FAZ in SCP in DRT Patient N.31, (**d**); shows FAZ in DCP in DRT as same patient in (**c**)

The central foveal thickness (CFT) differed significantly between the two groups (CME & DRT), being 441.116 ± 166.71, 325.64 ± 92.64 um respectively, with (p = 0.004) and parafoveal thickness (PFT) (426.80 ± 108.02, 371.83 ± 71.84, respectively, with p = 0.039). The FAZ was larger in the CME group, but without a statistically significant difference **(**Fig. 2).

**Fig. 3:**
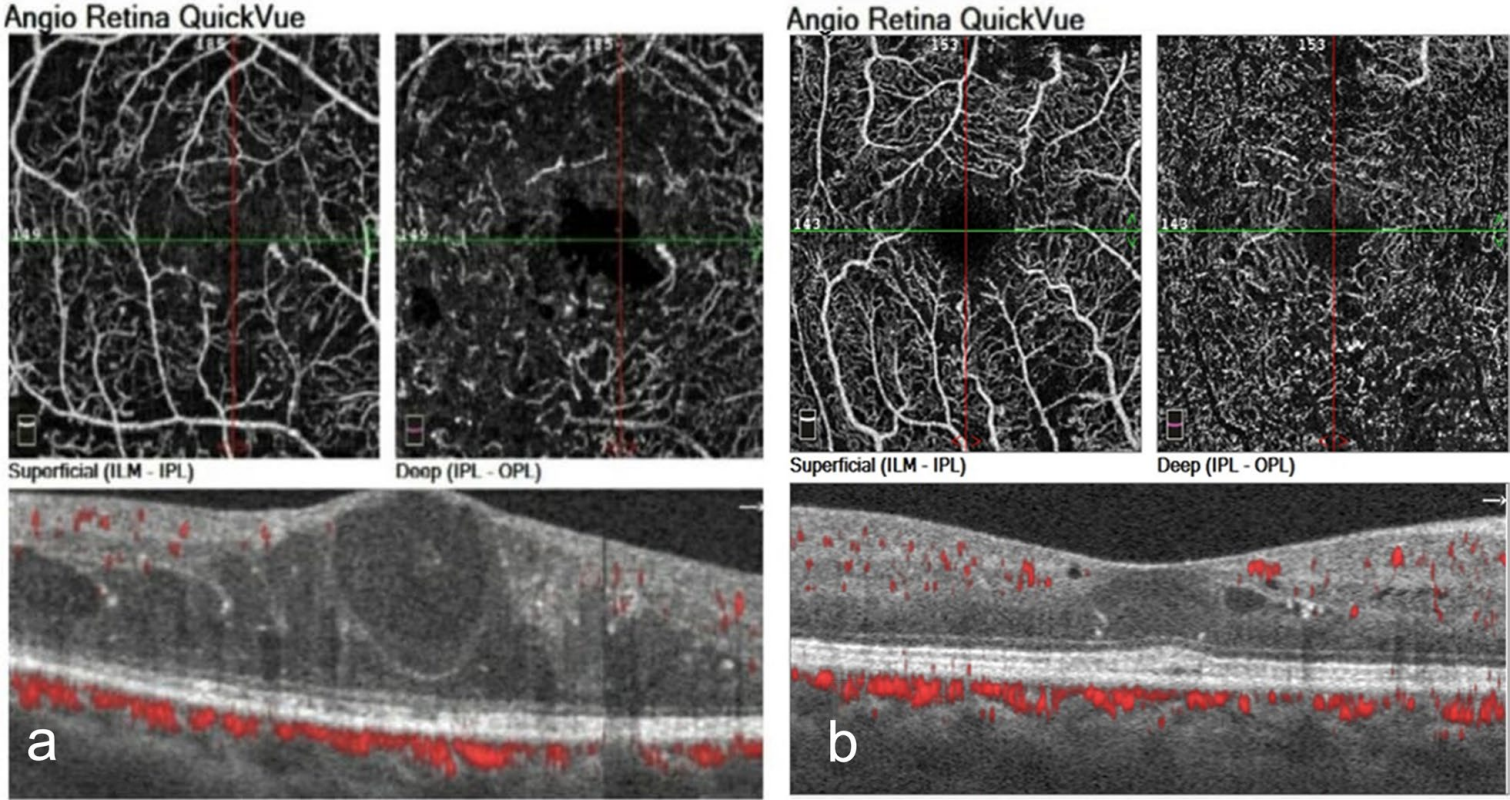
VD on 3 × 3 mm OCTA images in the different morphological patterns of DME **a** CME, patient N11 **b**; DRT, Patient N 36

**Fig. 4:**
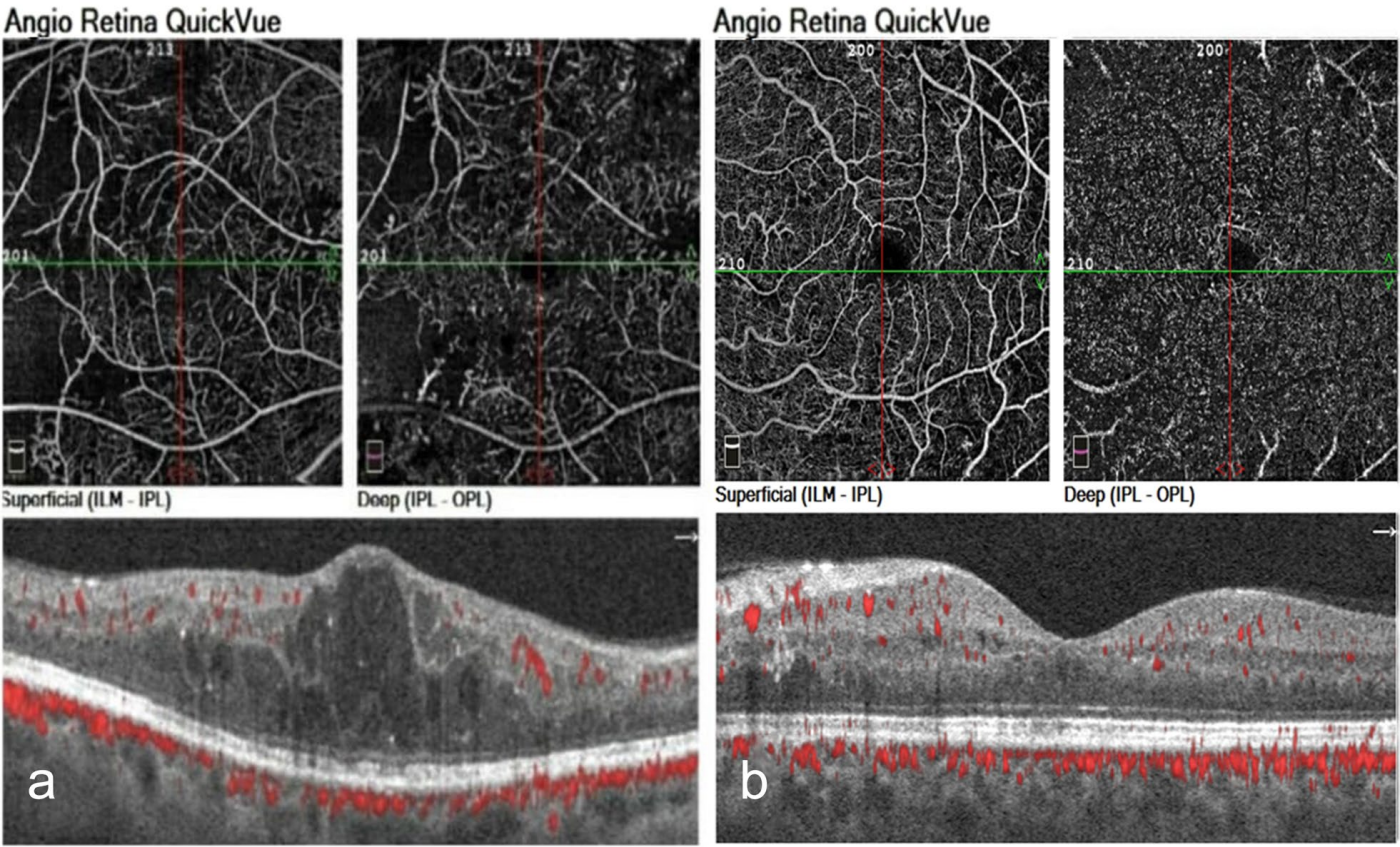
VD on 6 × 6 mm OCTA images in the different morphological patterns of DME (**a**) CME (**b**); DRT of the same patients in Fig. 2

There were no statistically significant differences in the mean vascular density (VD) between both groups in the 3 × 3 and 6 × 6 mm scans in either the SCP or DCP (Figs. 3, 4).

**Fig. 5:**
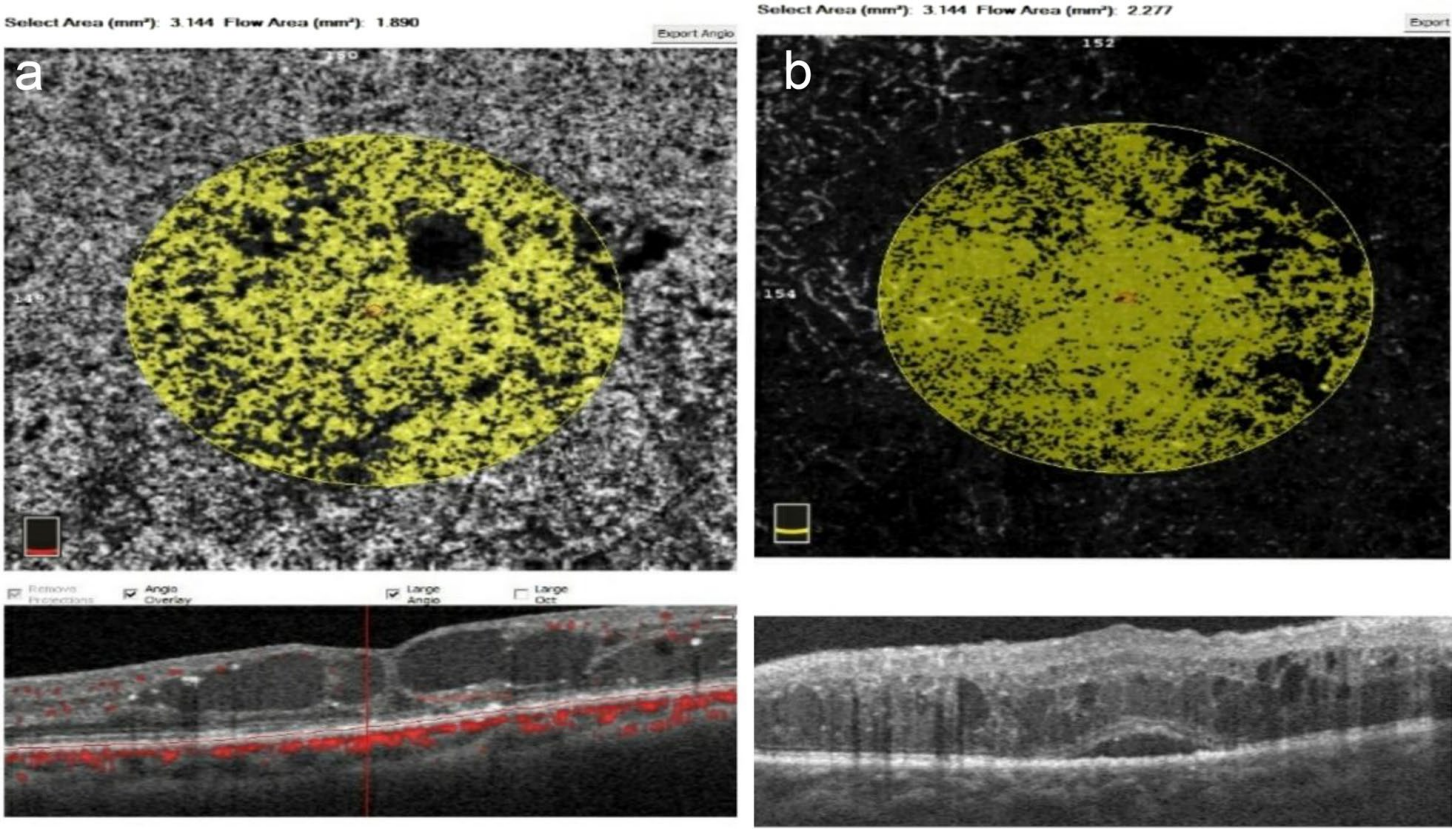
CC flow area (3.144 mm^2^) in OCTA images in the different morphological patterns of DME **a** in CME, **b** in DRT

Choriocapillaris (CC) flow was lower in the CME group compared to the DRT group but without a statistically significant difference (Fig. 5). OCTA characteristics of both groups are described in Table 3.

**Table 3:** OCTA characteristics of both groups (CME Vs DRT)

OCTA parameters	CME group	DRT group	P value
CFT			
3 × 3	441.12 ± 166.71	325.64 ± 92.64	0.004*
6 × 6	428.33 ± 142.44	314.12 ± 105.98	0.002*
PFT			
3 × 3	426.80 ± 108.02	371.83 ± 71.84	0.039*
6 × 6	426.04 ± 105.29	363.88 ± 80.01	0.021*
FAZ			
SCP	0.57 ± 0.28	0.53 ± 0.25	0.574
DCP	0.54 ± 0.29	0.50 ± 0.24	0.563
VD, SCP 3 × 3			
Whole image	36.43 ± 5.29	35.86 ± 3.62	0.657
Fovea	16.64 ± 6.00	16.64 ± 7.64	0.998
Parafovea	37.78 ± 6.65	37.35 ± 3.96	0.783
VD, SCP 6 × 6			
Whole image	41.34 ± 4.90	41.46 ± 4.14	0.925
Fovea	22.60 ± 7.92	19.26 ± 9.24	0.167
Parafovea	40.91 ± 5.47	41.03 ± 4.93	0.938
Perifoveal	41.78 ± 5.34	42.30 ± 4.17	0.698
VD, DCP 3X3			
Whole image	40.96 ± 5.29	40.45 ± 6.09	0.754
Fovea	27.16 ± 11.62	26.57 ± 9.08	0.843
Parafovea	41.80 ± 5.48	42.05 ± 6.59	0.884
VD, DCP 6 × 6			
Whole image	41.53 ± 4.54	40.51 ± 4.49	0.419
Fovea	30.59 ± 8.07	30.15 ± 9.11	0.856
Parafovea	46.19 ± 5.45	44.70 ± 5.36	0.326
CC			
3 × 3	1.74 ± 0.22	1.80 ± 0.22	0.311
6 × 6	1.79 ± 0.28	1.91 ± 0.17	0.062

*OCTA* Optical Coherence tomography angiography, *CME* Cystoid Macular edema, *DRT* Diffuse Retinal Thickening, *CFT* Central Foveal Thickness, *PFT* Parafoveal Thickness, *FAZ* Foveal Avascular Zone, *VD* Vascular density, *SCP* Superficial Capillary Plexus, *DCP* Deep Capillary Plexus, *CC* Choriocapillaris

*Highly significant comparison

### Comparison between cases with SRF (SRF + +) versus cases without SRF (SRF−−)

The lipid profile showed similar levels of total serum cholesterol, LDL and triglycerides with the only statistically significant difference being a higher level of LDL in the DRT/SRF +  + group compared to the DRT/SRF−− group (p = 0.033) (Tables 4 and 5).

**Table 4:** Comparison between cases with SRF Vs without SRF

	DME without SRF	DME with SRF	P value
Mean age (years)	56.91 ± 7.94	55.00 ± 7.59	0.804
BCVA (LogMAR)	0.75 ± 0.31	0.79 ± 0.26	0.672
HbA1c	9.43 ± 2.01	9.61 ± 1.46	0.386
Duration of DM (years)	10.45 ± 2.26	10.23 ± 2.57	0.854
KFTs			
Creatinine	1.04 ± 0.33	1.02 ± 0.23	0.581
Urea	39.74 ± 9.28	37.21 ± 11.09	0.546
Lipid profile			
Cholesterol	229.97 ± 51.86	243.75 ± 54.67	0.413
TCG	196.31 ± 126.56	181.85 ± 69.70	0.131
HDL	48.18 ± 8.35	42.98 ± 8.57	0.270
LDL	153.41 ± 53.71	175.70 ± 45.79	0.938
CFT			
3X3	360.84 ± 142.21	423.44 ± 147.05	0.528
6X6	360.34 ± 141.58	394.35 ± 131.94	0.856
PFT			
3X3	377.75 ± 86.69	437.67 ± 99.37	0.404
6X6	377.31 ± 86.34	426.30 ± 110.36	0.397
FAZ			
SCP	0.56 ± 0.23	0.54 ± 0.32	0.907
DCP	0.52 ± 0.25	0.52 ± 0.29	0.714
VD, SCP 3X3			
Whole image	35.82 ± 4.32	36.72 ± 4.86	0.352
Fovea	15.74 ± 6.37	18.24 ± 7.42	0.578
Parafovea	37.08 ± 5.49	38.43 ± 5.34	0.277
Temporal	36.07 ± 5.81	38.64 ± 6.39	0.258
Superior	37.31 ± 5.91	38.49 ± 6.52	0.126
Nasal	36.58 ± 5.16	37.51 ± 5.27	0.627
Inferior	39.13 ± 5.82	39.04 ± 6.86	0.528
VD, SCP 6 × 6			
Whole image	40.60 ± 4.76	42.69 ± 3.84	0.009*
Fovea	19.42 ± 8.67	23.50 ± 8.24	0.338
Parafovea	39.88 ± 5.43	42.72 ± 4.26	0.041*
Temporal	40.67 ± 6.06	43.61 ± 5.46	0.198
Superior	39.58 ± 6.25	42.46 ± 4.90	0.009*
Nasal	38.24 ± 7.69	41.96 ± 4.30	0.058
Inferior	40.65 ± 6.59	42.85 ± 4.80	0.097
Perifoveal	41.09 ± 5.24	43.53 ± 3.54	0.006*
VD, DCP 3X3			
Whole image	41.51 ± 5.85	39.27 ± 5.11	0.364
Fovea	24.03 ± 8.42	31.89 ± 11.67	0.066
Parafovea	42.70 ± 6.27	40.54 ± 5.36	0.239
Temporal	42.73 ± 7.17	39.61 ± 6.22	0.014*
Superior	43.28 ± 7.11	40.35 ± 7.41	0.407
Nasal	44.41 ± 6.63	41.49 ± 6.52	0.531
Inferior	43.62 ± 7.04	40.24 ± 5.32	0.070
VD, DCP 6 × 6			
Whole image	40.28 ± 4.93	42.26 ± 3.49	0.281
Fovea	29.00 ± 9.23	32.59 ± 6.84	0.753
Parafovea	45.22 ± 6.15	45.90 ± 4.06	0.813
Temporal	46.35 ± 6.51	47.08 ± 5.86	0.243
Superior	45.14 ± 6.47	45.29 ± 6.41	0.917
Nasal	45.65 ± 7.01	47.17 ± 4.30	0.641
Inferior	43.94 ± 8.05	44.42 ± 4.09	0.891
CC			
3X3	1.83 ± 0.22	1.66 ± 0.18	0.052
6X6	1.92 ± 0.17	1.73 ± 0.28	0.012*

*n* Number, *CME* Cystoid Macular edema, *DRT* Diffuse Retinal Thickening, *SRF* Subretinal Fluid, *SD* Standard Deviation, *DM* Diabetes mellitus, *BCVA* Best Corrected Visual Acuity, *HbA1C* Glycosylated Haemoglobin, *KFTs* Kidney Function Tests, *TCG* Triglycerides, *HDL* High density lipoproteins, *LDL* Low density lipoproteins, *OCTA* Optical Coherence tomography angiography, *CFT* Central Foveal Thickness, *PFT* Parafoveal Thickness, *FAZ* Foveal Avascular Zone, *VD* Vascular density, *SCP* Superficial Capillary Plexus, *DCP* Deep Capillary Plexus, *CC* Choriocapillaris

*Highly significant comparison

**Table 5:** Comparison of CME/SRF +  + Vs CME/SRF – & DRT/SRF +  + Vs DRT/SRF –

	CME/SRF−−	CME/SRF + +	P value	DRT/SRF−−	DRT/SRF + +	P value
HbA1c	9.14 ± 1.95	9.79 ± 1.66	0.386	9.75 ± 2.10	9.42 ± 1.30	0.659
Duration of DM (year)	10 ± 1.83	9.88 ± 2.78	0.980	10.80 ± 2.90	10.60 ± 2.29	0.849
KFTs						
Creatinine	1.11 ± 0.29	1.04 ± 0.28	0.581	0.97 ± 0.36	1.00 ± 0.17	0.779
Urea	39.32 ± 9.95	36.60 ± 13.04	0.546	40.21 ± 8.79	37.81 ± 9.43	0.522
Lipid profile						
Cholesterol	237.18 ± 54.90	221.20 ± 32.98	0.413	221.80 ± 48.75	266.30 ± 63.97	0.060
TCG	209.76 ± 120.91	146.00 ± 56.71	0.131	181.07 ± 135.23	217.70 ± 64.69	0.435
HDL	47.00 ± 7.16	43.57 ± 8.42	0.270	49.51 ± 9.60	42.40 ± 9.14	0.077
LDL	154.35 ± 55.60	152.80 ± 36.34	0.938	152.33 ± 53.41	198.60 ± 44.06	0.033*
CFT						
3X3	426.29 ± 159.97	472.63 ± 187.43	0.528	286.67 ± 66.62	384.10 ± 98.25	0.007*
6X6	424.41 ± 160.35	435.00 ± 113.25	0.856	287.73 ± 66.09	353.7 ± 142.32	0.196
PFT						
3X3	414.12 ± 99.56	453.75 ± 126.99	0.404	336.53 ± 43.43	424.80 ± 75.47	0.001*
6X6	412.59 ± 99.49	448.90 ± 116.23	0.397	337.33 ± 44.40	403.70 ± 105.20	0.086
FAZ						
SCP	0.58 ± 0.19	0.57 ± 0.40	0.907	0.54 ± 0.27	0.52 ± 0.24	0.815
DCP	0.53 ± 0.25	0.57 ± 0.36	0.714	0.52 ± 0.27	0.47 ± 0.21	0.632
VD, SCP 3X3						
Whole image	35.74 ± 4.58	37.90 ± 6.65	0.352	35.91 ± 4.16	35.78 ± 2.83	0.930
Fovea	16.16 ± 5.86	17.64 ± 6.58	0.578	15.25 ± 7.07	18.72 ± 8.36	0.275
Parafovea	36.76 ± 6.21	39.92 ± 7.47	0.277	37.43 ± 4.74	37.23 ± 2.62	0.906
VD, SCP 6X6						
Whole image	39.54 ± 4.83	44.42 ± 3.33	0.009*	41.80 ± 4.52	40.96 ± 3.66	0.629
Fovea	21.45 ± 8.21	24.54 ± 7.42	0.338	17.13 ± 8.88	22.46 ± 9.27	0.162
Parafovea	39.28 ± 5.27	43.69 ± 4.84	0.041*	40.55 ± 5.72	41.74 ± 3.58	0.566
Perifoveal	39.93 ± 5.58	44.92 ± 3.11	0.006*	42.41 ± 4.66	42.14 ± 3.53	0.879
VD, DCP 3X3						
Whole image	41.63 ± 5.76	39.53 ± 4.09	0.364	41.37 ± 6.16	39.07 ± 6.01	0.366
Fovea	24.24 ± 9.74	33.35 ± 13.49	0.066	23.80 ± 6.97	30.72 ± 10.61	0.060
Parafovea	42.70 ± 5.93	39.89 ± 4.03	0.239	42.71 ± 6.85	41.07 ± 6.39	0.554
VD, DCP 6X6						
Whole image	40.79 ± 4.94	42.78 ± 3.64	0.281	39.69 ± 5.02	41.73 ± 3.44	0.276
Fovea	30.20 ± 9.38	31.24 ± 5.56	0.753	27.63 ± 9.17	33.93 ± 7.99	0.091
Parafovea	46.39 ± 6.54	45.86 ± 3.08	0.813	43.89 ± 5.59	45.93 ± 5.03	0.361
Perifoveal	41.59 ± 5.45	44.19 ± 3.88	0.199	42.47 ± 8.00	42.41 ± 3.70	0.984
CC						
3 × 3	1.80 ± 0.22	1.62 ± 0.16	0.052	1.87 ± 0.21	1.70 ± 0.19	0.049*
6 × 6	1.89 ± 0.19	1.62 ± 0.33	0.012*	1.96 ± 0.15	1.84 ± 0.18	0.084

*n* Number, *CME* Cystoid Macular edema, *DRT* Diffuse Retinal Thickening, *SRF* Subretinal Fluid, *SD* Standard Deviation, *BCVA* Best Corrected Visual Acuity, *HbA1C* Glycosylated Haemoglobin, *KFTs* Kidney Function Tests, *TCG* Triglycerides, *HDL* High density lipoproteins, *LDL* Low density lipoproteins, *OCTA* Optical Coherence tomography angiography, *DM* Diabetes mellitus, *CFT* Central Foveal Thickness, *PFT* Parafoveal Thickness, *FAZ* Foveal Avascular Zone, *VD* Vascular density, *SCP* Superficial Capillary Plexus, *DCP* Deep Capillary Plexus, *CC* Choriocapillaris

*Highly significant comparison

When comparing both subtypes of CME in 6 × 6 mm images, VD in SCP was significantly lower in the CME/SRF−− group as compared to the CME/SRF +  + group in whole image, parafoveal and perifoveal regions with (P = 0.009, 0.041 and 0.006 respectively). Differences in the 3 × 3 images were not statistically significant, except in the temporal parafoveal DCP (Table 5).

As regards both subtypes of DRT in the 3 × 3 and 6 × 6 mm images, there were no statistically significant differences in VD between groups (Table 5).

The VD in all cases with SRF and cases without SRF in SCP & DCP in 3 × 3 mm OCTA imaging were similar*,* but on 6 × 6 mm OCTA imaging, statistically significant differences were found in the whole image VD (p = 0.0.009), parafovea (p = 0.041) and perifovea (p = 0.006) (Table 4).

On comparing CC flow area between the SRF-—and SRF +  + groups, a statistically significant difference was found only on 6 × 6 mm imaging; being 1.92 ± 0.17 & 1.73 ± 0.28 in respectively (p = 0.012) (Table 4).

CC flow area was also compared within each group on subdivision to: CME/SRF +  + /CME/SRF−− and DRT/SRF +  + /DRT/SRF−−. On 3 × 3 mm imaging; the only significant was found between the DRT/SRF– group was 1.87 ± 0.21 and in the DRT/SRF +  + group, it was 1.70 ± 0.19, (p = 0.049). On 6 × 6 imaging; the only significant difference in CC flow was between the CME/SRF−− group was 1.89 ± 0.19, and in the CME/SRF +  + group was 1.62 ± 0.33, (p = 0.012) (Table 5).

When comparing eyes with neurosensory detachment (NSD) and without NSD, the BCVA in LogMAR was not significantly different between both groups, where the SRF−− group had a mean BCVA (LogMAR) of 0.75 ± 31 and the SRF +  + was 0.79 ± 0.26 (p = 0.672). Therefore, the mere presence or absence of SRF was not a biomarker of visual function.

### Correlation with BCVA

Linear regression analysis was performed using variables significantly associated with logMAR BCVA in univariate analysis (HbA1c and triglycerides) and DME morphology. DRT morphology was most strongly associated with logMAR BCVA, as compared to other variables (p < 0.001). This indicated that DME morphology was the strongest predictor for BCVA. HbA1C and triglyceride levels were also significantly associated with logMAR BCVA in linear regression analysis (p = 0.033, 0.028) respectively. However, no significant correlation was found between BCVA and the OCTA parameters (3 × 3 & 6 × 6) in all patients.

### Correlation with the age

As regards the age, there was a significant negative correlation between the age with the foveal and parafoveal thickness in 3 × 3 mm OCTA imaging (r = −0.396 and p = 0.004, r = −0.319 and p = 0.024) respectively. Likewise, there was a significant negative correlation between the age and foveal and parafoveal thickness in 6 × 6 mm imaging (r = −0.340 and p = 0.014, r = −0.302 and p = 0.030) respectively.

### Correlation with the severity of the DR

There was a significant negative correlation between the severity of DR and foveal VD in SCP and DCP in 3 × 3 OCTA imaging (r = −0.292 and p = 0.04, r = −0.364 and p = 0.009) respectively. Furthermore, there was a significant positive correlation between severity of DR and FAZ in SCP 3 × 3 mm OCTA imaging (r = 0.481, p = 0.009).

### Correlation with the duration of diabetes

As regards to the correlation between the duration of diabetes and the OCTA parameters, we found a significant negative correlation in vascular density (VD) of the whole image in the SCP and DCP (on 6 × 6 imaging) in the CME group (r = −0.407, p = 0.035, r = −0.537 and p = 0.004 respectively). In addition, there was a significant negative correlation between the duration and the VD of the whole image in the DCP (on 6 × 6 imaging) in the DRT group (r = −0.559, p value 0.004).

On correlating the duration with cases of CME with SRF, we found a significant negative correlation in the VD of the whole image in the SCP and DCP (in the 6 × 6 scans, r = −0.620, p = 0.008 & r = −0.694, p = 0.002 respectively). In cases of CME without SRF, there was a highly significant negative correlation between the duration and CC flow (in the 6 × 6 scans, r = −0.844, p = 0.002).

In DRT with SRF, the only significant negative correlation was in the VD of the whole image DCP (in the 6 × 6 scans, r = −0.621, p = 0.014).

## Discussion

The OCT patterns of DME are prognostic factors in the response to various treatment modalities. However, a direct link between OCT pattern and treatment response is yet to be established. For example, it remains unknown why patients with CME may gain a greater improvement in visual and anatomical outcomes after administration of intravitreal bevacizumab injection as their primary treatment, in comparison to patients with DRT [6]. In contrast, Kim et al., reported that intravitreal injection of bevacizumab was more effective in the treatment of DRT type than in the SRF or CME types of DME [7]. These conflicting findings prompt further investigation by OCTA; to differentiate the microvascular features the subtypes of DME, provide insight into the underlying pathophysiology and thus assist in treatment decisions.

In our study, we found that there were no significant differences in the microvascular features as detected by OCTA between DME patients with CME and DRT. There was a significant difference in BCVA between the CME group (0.88 ± 0.29) and DRT group (0.64 ± 0.22) (p = 0.001).

The presence of SRF did not have a significant effect on BCVA in the CME subgroups or DRT subgroups. Also, when comparing all SRF−− cases versus all SRF +  + cases, the difference in mean LogMAR BCVA was not significant; indicating that the presence of SRF is not a biomarker of visual function.

Also, there were no significant correlations between BCVA and parameters of OCTA (3X3 & 6X6) in all patients or in subgroups of DME. Thus, the only predictor of visual acuity is the pattern of DME (CME or DRT); irrespective to presence of SRF, CFT, VD, FAZ or choriocapillaris vascular flow.

Similarly, Kang et al. [8], found that the best corrected visual acuity in the diffuse retinal thickening group is significantly better than in the cystoid macular edema group and the serous retinal detachment group. It was the poorest and central macular thickness was the highest in the CME pattern group [8]. Likewise, Acan et al. [9], reported that the BCVA was worse in the CME group [9].

Arf et al. [10], reported that BCVA was significantly different only in the group with cystoid macular degeneration compared with the groups with CME and diffuse edema. However, there was no association between BCVA and presence of subfoveal neurosensory detachment (SND), hard exudates, vitreomacular traction or epiretinal membrane [10].

Sharma et al., found that the baseline visual acuity and central macular thickness (CMT) of their DRT group were better than that of their other two groups (CME and SND) [11]. It was also found that increasing retinal thickness in all patterns was significantly correlated with worse visual acuity, but the association was significantly worse in their CME group than with DRT and SND [12].

Giocanti-Aurégan et al. [13], reported that similar BCVA was observed in both CME and DRT regardless of the presence of SND [13].

However, in contrast to what we concluded and to Giocanti-Aurégan et al., DME with SND was correlated with significant impairment in BCVA in Vujosevic et al. [14].

In our study, we found that CFT is not considered as a reliable indicator for visual acuity. No correlation was found between the BCVA and CFT (p = 0.196) or parafoveal thickness (PFT) (p = 0.329). Pelosini et al. [15], reported similar results; where it is always valuable to consider other associated biomarkers, like the pattern of DME, rather than the CFT [15].

In this study, the mean CC vascular flow was 1.77 + 0.55, but without a significant difference in CC flow area in the CME group or DRT group. The presence of SRF had a significant effect on CC flow in the 3 × 3 imaging in the DRT/SRF +  + subgroup versus DRT/SRF−− subgroup (p = 0.049) and in the 6 × 6 imaging in CME/SRF +  + subgroup versus CME/SRF−− subgroup (p = 0.012).

Likewise, Conti et al., reported a significant reduction of CC in diabetic patients compared to normal controls using SS-OCTA [16]. They concluded that decreased CC perfusion could be an early indicator of otherwise clinically undetectable diabetic vasculopathy [17].

In our study, the correlation between CC vascular flow and BCVA (p = 0.908), severity of DR, HTN, lipid profile, kidney functions and HBA1C was not found to be significant.

However, Gendelman et al. [18], reported that in diabetic eyes the CC flow impairment was related to the severity DR and with a greater regional impairment in middle and inner regions due to age and disease severity [18].

Our study also showed a significant correlation between BCVA and HBA1C in the cases, as one group (p = 0.033), but not on subdivision to DRT and CME groups. Different studies concluded contradictory results with respect to this association. For instance, our findings concur with those of Gupta et al. [19]. While another 2011 study demonstrated an association between high HbA1C levels and NSD [20]. Acan et al. [20], found (HbA1c) level was significantly higher in patients with the DRT pattern group than CME or SND groups [9].

A significant correlation was found between BCVA and TCG level (p = 0.028), but similar to other studies, we did not find a significant relationship between serum lipids and macular thickness or severity of edema. The lipid profile analysis showed that the only statistically significant parameter was LDL being significantly correlated with diffuse retinal thickening associated with SRF (p = 0.033).

Most lipid assays have not been consistently associated with DR or DME [21]. Triglyceride, HDL, VLDL, and hemoglobin levels were not found to be different between SRF +  + and SRF—groups [22].

We found a significant negative correlation between the severity of DR and foveal VD in SCP in 3 × 3 OCTA imaging, (p = 0.04), whole image VD in the DCP in 3 × 3 OCTA imaging, (p = 0.009) and the measured areas of the FAZ in the SCP in 3 × 3 OCTA imaging (p = 0.009).

Vujosevic et al., found a significant correlation between the severity of DR and foveal VD in SCP (p = 0.04), whole image VD in DCP (p = 0.009) in 3 × 3 OCTA imaging. So, as the DR became more severe, the reduction of VD was greater (PDR being associated with greater capillary dropout and thus, lower values of vascular density) [14].

Liu et al., also reported; they found that there was a significant correlation between vessel density in the SCP and DCP with increasing severity of DR [23]. It was also reported, that as the stage of DR progressed, the mean VD values decreased and FAZ area demonstrated the strongest inverse correlation with DR severity [24, 25].

In our study, the duration of diabetes was significantly negatively correlated with multiple OCTA parameters. While our findings, the longer the duration of the diabetes the lower the VD and the CC flow, is logical, other studies do not concur. They found that the duration of diabetes did not correlate with the BCVA, FAZ size or any of the OCTA parameters., which they justified that patient history is often unreliable patient history and that duration is only one of the contributing risk factors for DR [26, 27].

The limitations of our study are the absence of Type I diabetes mellitus patients and patients with nephropathy. We recommend larger studies, preferably including different types of DM patients (I DM & II DM), are needed to clarify the relationship between the numerous OCTA-derived vascular parameters and different clinical and laboratory parameters.

## Conclusion

The morphology of macular edema (DRT versus CME); irrespective of SRF presence, was the factor most predictive of BCVA in patients with DME. On the other hand, CMT did not correlate with logMAR BCVA and were no significant differences regarding OCTA findings in patients with both subtypes of DME (CME and DRT). CME subtype could be an independant predictor of poor BCVA in patients with DME.

## Data Availability

The datasets used and/or analysed during the current study available from the corresponding author (Lameece Hassan-lameecemoustafa@kasralainy.edu.eg) on reasonable request.
